# Ivy-Street Hospital

**Published:** 1886-01-20

**Authors:** 


### IVY-STREET HOSPITAL.

This Institution - the medical control of which is in charge of the Faculty of the Southern Medical College of Atlanta—is doing a good work for the sick, not only of Atlanta but of many from a distance. Professor Powell, the superintendent, with commendable zeal and with that broad liberality and kindliness of disposition for which he is noted, is giving the Institution his special supervision, while the members of the College Faculty are ever ready to give their cheerful and gratuitous aid in the treatment of the suffering inmates. Prompted by the double object of charity to the suffering and the benefit to the College, in the matter of furnishing bedside instruction to the medical class, Professor Powell, with the sanction of the Faculty, addressed a circular to the profession throughout the State, offering to accommodate any patient who, for the lack of facilities, the home physician might desire to send to the Institution for treatment. It is often the case, in village and country practice, that medical men, in a certain class of cases, are not prepared with suitable instruments and other facilities necessary for their proper treatment, and which facilities, indeed, they cannot always afford to furnish. The circular thus sent out has been favorably responded to—many expressing themselves as glad of the opportunity thus furnished them of sending such cases to a hospital conducted on principles of Charity, at which their patients, who have little or no means, can be scientifically treated and at mere nominal cost. The Southern Medical College of Atlanta is in a flourishing condition. Its present Class is large and intelligent. The Institution is, in all respects, well equipped, and the facilities for instruction unsurpassed by any school with which we are acquainted in this country.

